# Mapping global biodiversity connections with DNA barcodes: Lepidoptera of Pakistan

**DOI:** 10.1371/journal.pone.0174749

**Published:** 2017-03-24

**Authors:** Muhammad Ashfaq, Saleem Akhtar, Muhammad Athar Rafi, Shahid Mansoor, Paul D. N. Hebert

**Affiliations:** 1 Centre for Biodiversity Genomics, Biodiversity Institute of Ontario, University of Guelph, Guelph, Ontario, Canada; 2 National Institute for Biotechnology and Genetic Engineering, Faisalabad, Pakistan; 3 Insect Museum, National Agriculture Research Centre, Islamabad, Pakistan; Chang Gung University, TAIWAN

## Abstract

Sequences from the DNA barcode region of the mitochondrial COI gene are an effective tool for specimen identification and for the discovery of new species. The Barcode of Life Data Systems (BOLD) (www.boldsystems.org) currently hosts 4.5 million records from animals which have been assigned to more than 490,000 different Barcode Index Numbers (BINs), which serve as a proxy for species. Because a fourth of these BINs derive from Lepidoptera, BOLD has a strong capability to both identify specimens in this order and to support studies of faunal overlap. DNA barcode sequences were obtained from 4503 moths from 329 sites across Pakistan, specimens that represented 981 BINs from 52 families. Among 379 species with a Linnaean name assignment, all were represented by a single BIN excepting five species that showed a BIN split. Less than half (44%) of the 981 BINs had counterparts in other countries; the remaining BINs were unique to Pakistan. Another 218 BINs of Lepidoptera from Pakistan were coupled with the 981 from this study before being compared with all 116,768 BINs for this order. As expected, faunal overlap was highest with India (21%), Sri Lanka (21%), United Arab Emirates (20%) and with other Asian nations (2.1%), but it was very low with other continents including Africa (0.6%), Europe (1.3%), Australia (0.6%), Oceania (1.0%), North America (0.1%), and South America (0.1%). This study indicates the way in which DNA barcoding facilitates measures of faunal overlap even when taxa have not been assigned to a Linnean species.

## Introduction

Biodiversity inventories are a critical element of biogeographic analysis and have traditionally been based on morphological approaches [1–3]. However, molecular analysis [4] has the advantage of both revealing patterns of regional genetic divergence and allowing biodiversity comparisons at larger geographic and taxonomic scales [5,6]. DNA barcoding [7] has gained general acceptance for specimen identification and species discovery [8], resulting in the assembly of barcode records from nearly 500,000 animal species on the Barcode of Life Data Systems (BOLD) (www.boldsystems.org) [9]. The use of DNA barcoding to examine genetic patterns [10] and to reveal biodiversity overlap in insect communities [11] is well documented. However, the implementation of the Barcode Index Number (BIN) system [12] as a proxy for species [13] is allowing DNA barcoding to support biodiversity assessment [14], providing the opportunity to accelerate biotic inventories [15–20]. As a consequence, BINs have already been used to examine animal biodiversity [21,22] at regional [23] and global [24] scales.

The order Lepidoptera has seen particularly intensive barcode analysis, work which has employed BINs to identify species [25], to analyze genetic structure [26], to discover regional species connections [27] and to explore biodiversity [28,29]. For example, Hausmann *et al*. [26] employed BINs to reveal genetic patterns in European geometrids, while Janzen *et al*. [30] used them to evaluate the diversity of skipper butterflies in Costa Rica. Zenker *et al*. [22] used BINs to examine the diversity of tiger moths in the Neotropics while Kekkonen & Hebert [31] used them to expose cryptic species complexes in Australian moths. These studies have not only validated the utility of BINs in biodiversity analysis [32], but have also expanded the barcode reference library for Lepidoptera which now includes one million records representing more than 116,000 BINs from 203 countries and dependent territories. As such, the Lepidoptera represent nearly a fourth of the 492,000 BINs on BOLD (accessed 23 December, 2016).

Global environmental change [33] and expanding human-mediated disturbance [34] have created the need for a better understanding of biodiversity connections at broad scales [35]. The growing volume of DNA barcode data can address this need. The current study employs the Lepidoptera of Pakistan as a model to show how DNA barcoding can both expose regional biodiversity and enable the estimation of faunal overlap with other countries.

## Materials and methods

### Ethics statement

No specific permissions were required for this study. Insects from private lands were collected only after the consent of the respective owners. The study did not involve endangered or protected species.

### Collections

**Fig 1:**
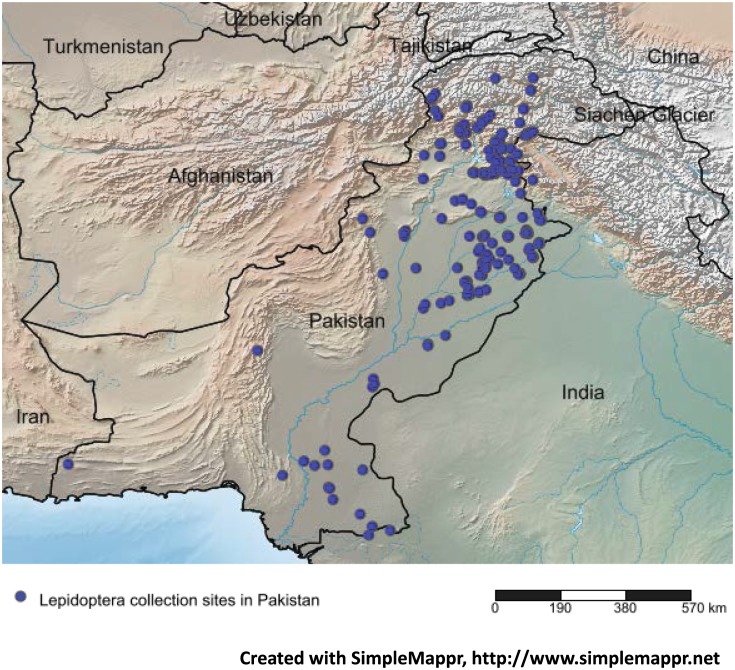
Map of Pakistan showing collection localities for specimens examined in this study.

Specimens were collected at 329 sites across Pakistan (Fig 1) ranging in elevation from 17m to 4283m. Diverse environments were sampled from deserts in the south to agricultural lands in the central regions and forests in the northwest. Collections were made from 2010–2013 with light traps, UV illuminated sheets, Malaise traps, sweep nets and hand collections (employed for larvae). Specimens were killed in cyanide jars, and placed individually in paper envelopes before specimens were relaxed, pinned, labeled and stored in the collection at the Insect Molecular Biology Laboratory at the National Institute for Biotechnology and Genetic Engineering (NIBGE), Faisalabad. Tissue samples from identified specimens were also donated by the National Insect Museum at the National Agriculture Research Centre (NARC), by the University of Agriculture, Faisalabad, and by several amateur collectors.

### Specimen identification

Morphological identifications were performed at the NARC, at the Insect DNA Barcoding Museum (NIBGE), and at the Centre for Biodiversity Genomics (CBG) with input from several taxonomic experts (see acknowledgements). Where possible, morphological identifications were supplemented by matching barcode records from the Pakistan specimens with those already on BOLD. Specimen information, collection data, and specimen images were submitted to BOLD and are available under the dataset, DS-MAMOT which can be accessed through the following DOI (dx.doi.org/10.5883/DS-MAMOT).

### Molecular analysis

A single leg was removed from each specimen with a sterile forceps and transferred to a 96-well microplate pre-loaded with 30 μl of 95% EtOH in each well. DNA extraction, PCR amplification and sequencing were performed at the Canadian Centre for DNA Barcoding (CCDB) following standard protocols (http://ccdb.ca/resources.php). PCR amplification of COI-5′ was performed with C_LepFolF and C_LepFolR (http://www.ccdb.ca/docs/CCDB_PrimerSets.pdf) primers. These primers are mixtures of LepF1 [36] /LCO1490 [37] and LepR1 [36] /HCO2198 [37], respectively. Amplifications involved 12.5 μL reactions containing standard PCR ingredients [38] and 2 μL of DNA template. PCR products were analyzed on a 2% agarose E-gel^®^ 96 system (Invitrogen Inc.) and amplicons were sequenced using the BigDye Terminator Cycle Sequencing Kit (v3.1) on an Applied Biosystems 3730XL DNA Analyzer. Sequences were assembled, aligned and edited using CodonCode Aligner (CodonCode Corporation, USA) before translation in MEGA5 [39] to verify that they were free of stop codons before submission to BOLD. All sequences generated in this study are accessible on BOLD in the dataset DS-MAMOT which also provides GenBank accession numbers.

### Data analysis

ClustalW nucleotide sequence alignments [40], genetic divergence, and neighbor-joining (NJ) clustering analysis were performed with MEGA5. The Kimura-2-Parameter (K2P) [41] distance model was used, along with pairwise deletion of missing sites, with nodal support estimated using 500 bootstrap replicates. Sequences meeting the quality criteria (>507 bp, <1% Ns, no stop codon or contamination flag) were assigned BINs by the Refined Single Linkage (RESL) algorithm implemented on BOLD [12]. The RESL algorithm runs monthly on all eligible sequences in BOLD and the resulting BIN array is accessible through individual BIN pages. The presence or absence of a “barcode gap” [42] was determined for each species to test the reliability of its discrimination. Using the "Barcode Gap Analysis" (BGA) tool on BOLD, a species is unambiguously identifiable if its maximum intraspecific distance is less than its Nearest-Neighbor (NN) distance. The relationship between geographic distance (km) and intraspecific distance (K2P) was analyzed for each species with at least three individuals and three locations using the “Geo-Distance Correlation” tool on BOLD that employs two methods to facilitate analysis. The Mantel Test [43] is used to examine the significance of the relationship between the geographic distance and genetic divergence matrices on a species-by-species basis. The second analysis examines the relationship across all species by employing the Minimum Spanning Tree for the collection sites as a proxy for the geographic distribution of each species, creating a set of values (one for each species) which is regressed against the corresponding set of maximum intraspecific sequence divergences at COI [44]. Geographic and genetic distances for all species were subsequently pooled in a single analysis enabling a scatter-plot of the two parameters with regression trendline generated in Excel.

BOLD was also searched for additional BINs of Lepidoptera from Pakistan, and the resultant 218 BINs were added to the 981 from this study before ascertaining BIN overlap with other countries. To permit this analysis, the 203 countries/ dependent territories with records were each assigned to a continent (Africa, Asia, Australia, Europe, North America, South America, Oceania) before BIN overlap was calculated in Excel and plotted using SimpleMappr (http://www.simplemappr.net/).

## Results

Nearly half of the specimens (2116/4503) (47%) were identified to a Linnaean species (by morphology) while another 1280 (28%) were placed in a genus. With one exception, the remaining 1107 (25%) specimens could be assigned to a family (S1 Table). Collectively, the specimens belong to 52 families, 108 subfamilies, 412 genera, 379 species and a total of 981 BINs (S1 Table). Most BINs (711/981) were represented by two or more individuals, but 270 were singletons (S2 Table). Table 1 lists the 52 families together with their specimen and BIN counts as well confamilial divergences (K2P). The Erebidae and Noctuidae possessed the largest number of specimens (1065, 830 respectively) and the most BINs (198, 168). By contrast, 29 families were represented by less than 10 specimens each, and eight by just a single specimen.

**Table 1:** Barcode index numbers and sequence divergence values (K2P) for the DNA barcode of COI for 52 families of moths collected in Pakistan. Sequence divergences are only reported for families with more than one BIN.

Family	Specimens	BINs	Max. (mean) divergence	Family	Specimens	BINs	Max. (mean) divergence
Adelidae	1	1	NA	Lecithoceridae	35	11	17.4 (9.8)
Autostichidae	31	2	9.6 (4.4)	Limacodidae	30	13	17.2 (10.4)
Batrachedridae	1	1	NA	Lyonetiidae	1	1	NA
Bedelliidae	4	4	15.2 (8.1)	Nepticulidae	1	1	NA
Blastobasidae	14	2	12 (5.2)	Noctuidae	830	168	18.1 (10)
Bombycidae	6	3	14.6 (9.6)	Nolidae	61	21	15.7 (10)
Brachodidae	4	2	6.3 (3.1)	Notodontidae	66	24	21.5 (13.5)
Brahmaeidae	2	2	8.3 (8.3)	Oecophoridae	7	5	17.8 (13)
Carposinidae	2	1	NA	Opostegidae	2	1	NA
Coleophoridae	7	2	9.4 (3.8)	Plutellidae	6	2	12.6 (4.6)
Cosmopterigidae	479	14	17.3 (5.8)	Psychidae	3	3	18.7 (17.9)
Cossidae	59	14	20.4 (13.8)	Pterophoridae	5	2	16.7 (6.7)
Crambidae	395	84	23 (12.1)	Pyralidae	181	62	19.9 (12.1)
Depressariidae	7	6	18.2 (12.7)	Saturniidae	19	3	13.5 (7.5)
Drepanidae	34	5	12.6 (6.6)	Scythrididae	6	5	15.9 (8.8)
Elachistidae	5	4	14.8 (12.4)	Sesiidae	1	1	NA
Erebidae	1065	198	25.1 (12.6)	Sphingidae	238	41	17.4 (10.8)
Eriocottidae	52	5	11.2 (6.2)	Stathmopodidae	6	2	16.4 (8.6)
Eupterotidae	9	3	18.1 (10)	Thyrididae	2	2	10.1 (10.1)
Euteliidae	39	5	10.4 (5.7)	Tineidae	37	15	21.7 (12.4)
Galacticidae	1	1	NA	Tortricidae	133	33	21.6 (11.2)
Gelechiidae	114	29	19.1 (11)	Uraniidae	6	2	11.2 (3.7)
Geometridae	433	154	20.1 (12.9)	Xyloryctidae	2	2	16.1 (16.1)
Gracillariidae	9	6	18.9 (14.5)	Yponomeutidae	14	3	10.8 (3.5)
Hyblaeidae	2	1	NA	Ypsolophidae	1	1	NA
Lasiocampidae	34	7	16.2 (10.1)	Zygaenidae	1	1	NA

**Fig 2:**
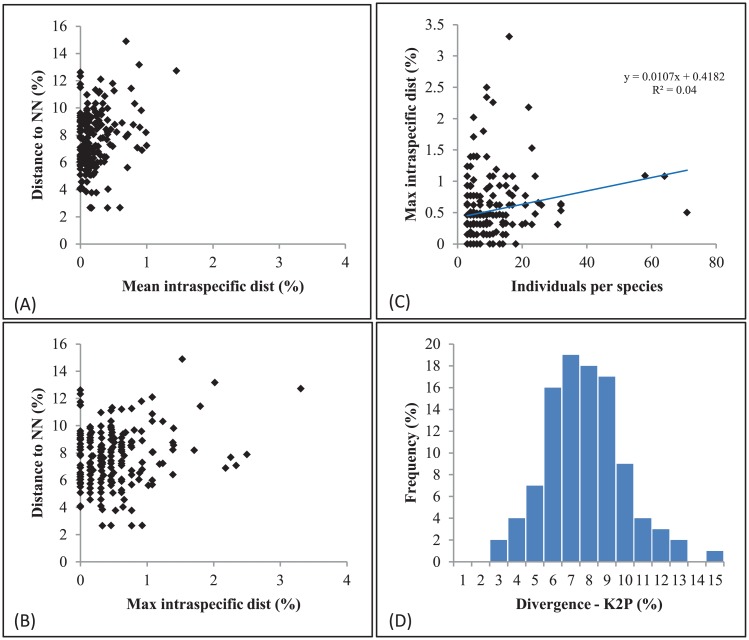
Barcode gap analysis for species of moths with three or more specimens collected in Pakistan. NN = nearest neighbor.

Intraspecific distances and BIN assignments for the 379 named species were calculated for the specimens from Pakistan (S3 Table). A third (35.6%) were represented by a single specimen while the rest had two or more records with *Cnaphalocrocis medinalis* having the highest count (71). These 379 species were assigned to 382 BINs. All species with multiple records were placed in a single BIN, barring *Amyna axis*, *Bedellia somnulentella*, *Biston suppressaria*, *Chiasmia hebesata*,and *Theretra alecto*, which were assigned to two, while two pairs of closely allied sphingids (*Hyles chuvilini—H*. *stroehlei*; *Hippotion boerhaviae*—*H*. *rosetta*) were each assigned to a single BIN. The species-BIN association was further supported by NJ clustering (S1 Fig). Excluding six species, *Nebrarctia transversa* (2.2%), *Amyna axis* (2.3%), *Theretra alecto* (2.3%), *Odontopera muscularia* (2.3%), *Diaphania indica* (2.5%), and *Scirpophaga excerptalis* (3.3%), the maximum intraspecific distance for all species was less than 2%. The maximum intraspecific distance for each species was lower than its NN distance (Fig 2A and 2B) and these values did not increase significantly with sample size (Fig 2C). However, as expected, divergences increased with taxonomic rank (Table 2) as conspecific divergences averaged 0.2% (range 0.0–3.3%) while congeneric (interspecific) (mean = 6.4%; range 2.7–16%) and confamilial (intergeneric) (mean = 11.3%; range 4.1–22.1%) were much higher. The inclusion of taxa unidentified to a species increased the mean confamilial divergence for 13 families and extended its range in the Crambidae and Erebidae (Table 1). When records from Pakistan were combined with those for conspecifics from other countries, there was more evidence of deep divergence (S3 Table). Intraspecific distances exceeded 2% in 89 species with particularly high values in *Coleophora trifolii* (K2P = 16.7%) and *Emmelina monodactyla* (K2P = 16.3%). Similarly, the incidence of BIN splits increased with 59 species assigned to two BINs, 19 to three BINs, 5 to four BINs, and one species (*Glyphodes onychinalis*) to six BINs.

**Table 2:** K2P sequence divergence in the COI barcode region for moth species from Pakistan with more than three specimens, genera with three or more species, and families with three or more genera. This analysis only considers specimens that were assigned to a Linnaean species.

Distance class	*n*	Taxa	Comparisons	Min (%)	Mean (%)	Max (%)
Intraspecific	1859	183	16929	0	0.2	3.3
Congeners	547	27	4120	2.7	6.4	16.0
Confamilial	1783	13	270379	4.1	11.3	22.1

**Fig 3:**
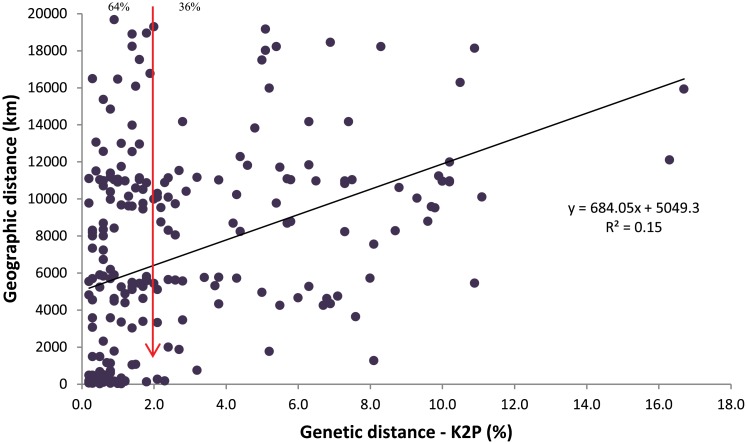
Intraspecific variation (K2P) at the COI gene against geographical extent (km) of moth species from Pakistan and their conspecifics from other regions. Vertical downward arrow (red) indicates the percentage of species with less or more than 2% divergence across their range.

S3 Table reports the results from the Geo-Distance-Correlation analysis for the 237 species with more than three barcode records. The K2P distances for conspecifics from different regions ranged from 0.2% - 16.7% while geographic distances between collection sites ranged from 23–19,665 km. The Mantel Test (R^2^) for 148 of these species was <0.5, indicating no significant relationship between genetic and geographic distances (S3 Table). However, the scatter-plot of the pooled geographic and genetic distances for all 237 species indicated a weak relationship (R^2^ = 0.15) between the two measures (Fig 3).

**Fig 4:**
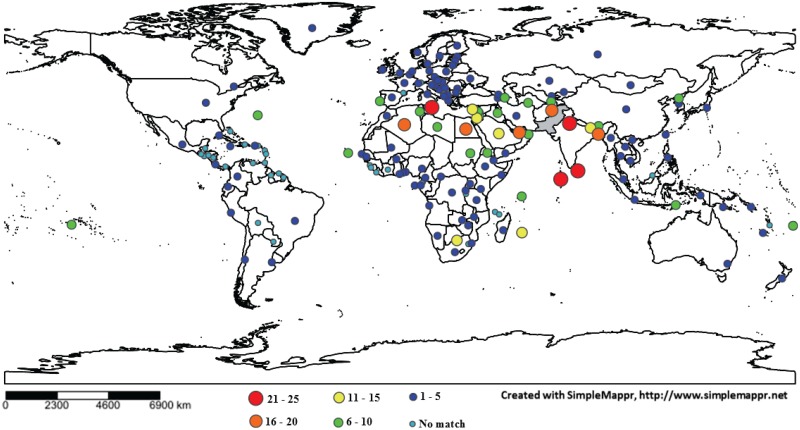
Heat map showing the percentage of Lepidoptera BINs from Pakistan (shaded gray) shared with other countries. Sharing (range) with other countries is colour-coded with varying circle sizes.

About 44% (427/981) of the BINs from Pakistan were shared with other countries while the remaining so far only possess records from this nation (S2 Table). Among the 1199 BINs of Pakistan Lepidoptera (218 from prior studies + 981 from this study), there was 21% overlap with the 856 BINs from India (21% of 856 BINs), Sri Lanka (21% of 86) or United Arab Emirates (20% of 436) and with other Asian nations (2.1% of 22,657). BIN sharing was far lower with nations on other continents including Africa (0.6% of 18,665), Australia (0.6% of 15,028), Europe (1.3% of 8,126), North and Central America (including the Caribbean) (0.1% of 31,283), Oceania (1.0% of 5189), and South America (0.1% of 19,863). Fifty-seven countries/ dependent territories, five with many (>500) BINs of Lepidoptera, did not share any species with Pakistan (Fig 4; S4 Table).

## Discussion

This study represents the first step toward the development of a DNA barcode reference library for the moths of Pakistan based upon the analysis of 4503 specimens which included representatives of 981 BINs (= proxy for species) from 52 families. Although about 300 species of butterflies are known from Pakistan [27,45,46], diversity estimates are only available for a few moth families [47–49]. As a first approximation, we hypothesize that about 8,000–10,000 species of Lepidoptera occur in Pakistan so barcode records cover just a tenth of the fauna. Among the 134 recognized families of moths ([50], www.boldsystems.org/), 52 were represented in this study. The Erebidae and Noctuidae dominated the collections with both the highest number of samples and BINs, an expected result since along with Geometridae [51] these two families are known for their species richness [52]. Only 47% of the specimens could be identified to a valid species underscoring the challenge in using traditional taxonomic approaches to evaluate biodiversity in regions that lack well-developed taxonomic resources.

Intraspecific divergences were greater than 2% in just six of the 244 named species represented by two or more specimens. Even including these six taxa with deep intraspecific divergence, conspecific divergence was always less than the NN distance. This result mirrors that from other large-scale analyses on Lepidoptera [53]. For example, Hebert et al. [54] found that only 9 of 1327 species from eastern North America showed intraspecific divergences above 1.5%. Another study which examined 1004 species from two distant European sites (1600 km apart) found that most (88%) of the shared species showed less than 2% maximum divergence [25].

The BIN system supported the genetic distinctiveness of the moth species from Pakistan as it assigned the 379 identified species to 382 BINs, mirroring results for the butterflies of this nation as 79 of 81 species were assigned to a unique BIN [27]. Similar congruence between BINs and morphological species has also been established in other studies on Lepidoptera [26]. For example, 90% of 92 Gelechiinae species from Finland [55] and 94% of 286 species of tiger moths from Brazil [22] were congruent with BINs. A similar pattern was reported for 3514 species of central European beetles as there was a 92% correspondence between morphospecies and BINs [56].

The integration of sequences from Pakistan with those from conspecifics from other regions substantially increased the frequency of deep sequence divergence; 89 species showed more than 2% intraspecific (maximum) distance and BIN splits were detected in 84 species. In fact, intraspecific divergence reached 16.7% in *Coleophora trifolii*, while specimens of *Glyphodes onychinalis* were assigned to six BINs. Prior studies have also reported cases of high intraspecific divergence and resultant BIN splits in Lepidoptera [57,58]. For example, 8% of the 1541 species of Noctuoidea analyzed from sites across Canada showed >2% intraspecific divergence and a similar incidence of BIN splits [13]. Likewise, 12.4% of European lepidopterans (124/1004) possessed more than 2% sequence divergence at COI [25]. Such cases may often reflect overlooked cryptic species [36], or simply misidentifications but this can only be confirmed by detailed taxonomic studies [59]. The coupling of cryptic species complexes with the prevalence of misidentified specimens represent important barriers to the analysis of species overlap at larger geographic scales.

Prior barcode studies have also revealed cases where different species show such low divergence that they are assigned to the same BIN [13,25,26]. In the present study, just two species pairs, *Hyles chuvilini*—*H*. *stroehlei* and *Hippotion boerhaviae*—*H*. *rosetta*, fell into this category. By comparison, Zahiri et al. [13] found that 21 species of 1541 Canadian noctuoid species shared their BIN with at least one other species, while Huemer et al. [25] found that 16 of 1004 lepidopteran species in Europe shared the BINs.

Although a study on one genus of aquatic beetles suggested that geographic variation in barcode sequences represents an important complication for barcode analysis [60], other investigations have not supported this conclusion. In their study on 1004 species of European Lepidoptera, Huemer et al. [25] found an increase in intraspecific divergence with distance, but it was so small in relation to NN distances that it rarely compromised identifications. A weak relationship between geographical distance and intraspecific divergence was also noted in the present study, as in earlier work on the butterflies of Pakistan, but again species assignments were not impeded [27]. As a consequence, it seems fair to conclude that while increased geographic coverage narrows the barcode gap, it rarely complicates species discrimination [61,62].

The order Lepidoptera includes about 180,000 described species with perhaps another 300,000 awaiting description [50,51]. Because barcode coverage is now available for 116,000 BINs, this order provides a good opportunity to evaluate how DNA barcoding can aid understanding of global biodiversity patterns. When distribution patterns for the 1199 BINs of this order from Pakistan were compared with all records from other regions, they indicated strong regional endemism as 21% of the BINs were shared with India and 2.1% with Asia while overlap with the faunas on other continents was just 0.1–1.3%. This result points towards a stronger regional biodiversity divergence, a conclusion supported by other studies [27,63]. However, the presence of unreported conspecifics in adjoining nations (Afghanistan, China, India, Iran) whose faunas have seen little barcode analysis may alter this assertion. Biodiversity loss [34] and the slow pace of conventional approaches to the documentation of the global fauna have created an urgent need for robust, rapid approaches for biodiversity analysis. The BIN system represents a partial solution as it circumvents the taxonomic impediment [12,19], enabling the automated comparisons of faunal overlap [24]. When coupled with access to low-cost sequencing methods [64], DNA barcoding represents an enabler for the biodiversity assessments needed to support conservation programs [65].

## Supporting information

S1 Fig NJ analysis of moth species from Pakistan based on the analysis of 2116 COI sequences derived from 379 species. Bootstrap values (>50%) (500 replicates) are shown above the branches. The scale bar shows K2P distances. The node for each species with multiple specimens is collapsed to a vertical line or triangle, with the horizontal depth indicating the level of intraspecific divergence. Analyses were conducted in MEGA5.
(EPS)

S1 Table Records for moths from Pakistan with BINs and morphological identification. (XLS)

S2 Table Unique (Pakistan-only), non-unique (shared with other countries) and singleton Lepidoptera BINs from Pakistan. (XLS)

S3 Table Barcode Index Numbers (BINs), maximum intraspecific distances (K2P) and geo-distance correlation analysis for the identified species of moths from Pakistan and their conspecifics from other countries with public records on the Barcode of Life Data Systems (www.boldsystems.org). (DOCX)

S4 Table Number of Lepidoptera BINs from other countries and continents shared with Pakistan. (XLS)
