# Supplementary material for: Mapping global biodiversity connections with DNA barcodes: Lepidoptera of Pakistan
Source: PLoS One. 2017 Mar 24;12(3):e0174749. doi: 10.1371/journal.pone.0174749 (PMC5365146; doi:10.1371/journal.pone.0174749)
Supplement: S3 Table — (DOCX) [file pone.0174749.s004.docx]

Table S3. Barcode Index Numbers (BINs), maximum intraspecific distances (K2P) and geo-distance correlation analysis for the identified species of moths from Pakistan and their conspecifics from other countries with public records on the Barcode of Life Data Systems ([www.boldsystems.org](http://www.boldsystems.org)).

| Family | Species | Intraspecific distance (individuals) | | BINs | | Geo. dist. (km) | M-R^2^ | M-*P* |
| --- | --- | --- | --- | --- | --- | --- | --- | --- |
|  |  | Pak | Comb | Pak | Comb |  |  |  |
| Bedelliidae | *Bedellia somnulentella* | 2 (2) | 10.9 (38) | 2 | 4 | 18132 | 0.9 | 0.01 |
| Blastobasidae | *Neoblastobasis eurotella* | 0.9 (10) | N/A | 1 | N/A | N/A | N/A | N/A |
| Brachodidae | *Phycodes minor* | 0 (3) | N/A | 1 | N/A | N/A | N/A | N/A |
| Brahmaeidae | *Brahmaea hearseyi* | N/A (1) | N/A | 1 | N/A | N/A | N/A | N/A |
|  | *Brahmaea wallichii* | N/A (1) | N/A | 1 | N/A | N/A | N/A | N/A |
| Carposinidae | *Epicopistis pleurospila* | 0.3 (2) | 11.1 (6) | 1 | 2 | 10107 | 0.0 | 0.8 |
| Coleophoridae | *Coleophora alcyonipennella* | N/A (1) | 0.3 (5) | N/A | 1 | 16481 | 0.4 | 0.08 |
|  | *Coleophora trifolii* | 0 (5) | 16.7 (60) | 1 | 1 | 15924 | 0.4 | 0.01 |
| Cosmopterigidae | *Anatrachyntis simplex* | N/A (1) | N/A | 1 | N/A | N/A | N/A | N/A |
|  | *Labdia stibogramma* | 0.6 (32) | N/A | 1 | N/A | N/A | N/A | N/A |
| Crambidae | *Achyra coelatalis* | 0 (2) | 0.3 (11) | 1 | 1 | 8304 | 0.0 | 0.55 |
|  | *Antigastra catalaunalis* | N/A (1) | 1.3 (8) | 1 | 1 | 10132 | 0.1 | 0.01 |
|  | *Aporodes floralis* | 0.8 (7) | 0.8 (9) | 1 | 1 | 3569 | 0.1 | 0.73 |
|  | *Autocharis fessalis* | 0.7 (7) | N/A | 1 | N/A | N/A | N/A | N/A |
|  | *Botyodes diniasalis* | 0.3 (3) | 0.3 (3) | 1 | 1 | 384 | 1.0 | 0.36 |
|  | *Bradina admixtalis* | N/A (1) | 4.3 (11) | 1 | 2 | 10226 | 1.0 | 0.01 |
|  | *Bradina diagonalis* | 0 (13) | N/A | 1 | N/A | N/A | N/A | N/A |
|  | *Chilo infuscatellus* | 0.8 (5) | 0.8 (5) | 1 | 1 | 202 | 0.1 | 0.89 |
|  | *Chilo partellus* | 0.6 (17) | 0.6 (19) | 1 | 1 | 465 | 0.0 | 0.26 |
|  | *Cirrhochrista annulifera* | 0.3 (3) | 9.3 (8) | 1 | 3 | 10041 | 0.0 | 0.67 |
|  | *Cnaphalocrocis medinalis* | 0.5 (71) | 9.9 (83) | 1 | 2 | 11236 | 0.0 | 0.09 |
|  | *Conogethes punctiferalis* | 0.9 (4) | 5.7 (25) | 1 | 3 | 11076 | 0.7 | 0.01 |
|  | *Cydalima perspectalis* | N/A (1) | N/A | 1 | N/A | N/A | N/A | N/A |
|  | *Diaphania indica* | 2.5 (9) | 2.5 (26) | 1 | 1 | 11136 | 0.4 | 0.01 |
|  | *Elophila difflualis* | N/A (1) | 10.2 (13) | 1 | 2 | 10919 | 0.0 | 0.32 |
|  | *Euchromius ocellea* | 0.6 (17) | 1.4 (111) | 1 | 1 | 13977 | 0.0 | 0.03 |
|  | *Euchromius ramburiellus* | 0 (2) | N/A | 1 | N/A | N/A | N/A | N/A |
|  | *Euclasta maceratalis* | N/A (1) | 0.8 (8) | 1 | 1 | 10377 | 0.1 | 0.17 |
|  | *Glyphodes onychinalis* | N/A (1) | 7.5 (14) | 1 | 6 | 11036 | 0.0 | 0.02 |
|  | *Herpetogramma licarsisalis* | 0.3 (9) | 6.3 (108) | 1 | 3 | 14166 | 0.0 | 0.87 |
|  | *Herpetogramma stultalis* | N/A (1) | 7.3 (5) | 1 | 2 | 10826 | 0.0 | 0.48 |
|  | *Hodebertia testalis* | 0.3 (2) | 1.6 (11) | 1 | 3 | 11136 | 0.4 | 0.01 |
|  | *Hydriris ornatalis* | 0.2 (4) | 8.7 (6) | 1 | 3 | 8284 | 0.4 | 0.03 |
|  | *Lamprophaia ablactalis* | 0.2 (2) | 9.7 (12) | 1 | 2 | 9577 | 0.0 | 0.36 |
|  | *Leucinodes orbonalis* | 1.2 (2) | 6.3 (25) | 1 | 3 | 11832 | 0.7 | 0.01 |
|  | *Loxostege nudalis* | 1.4 (6) | 1.4 (6) | 1 | 1 | 1045 | 0.1 | 0.64 |
|  | *Maruca vitrata* | 0.2 (2) | 8.3 (27) | 1 | 4 | 18223 | 0.0 | 0.29 |
|  | *Nacoleia commixta* | N/A (1) | N/A | 1 | N/A | N/A | N/A | N/A |
|  | *Nausinoe geometralis* | N/A (1) | 4.2 (5) | 1 | 2 | 8684 | 1.0 | 0.21 |
|  | *Nomophila noctuella* | 0.3 (31) | 5.1 (71) | 1 | 2 | 19166 | 0.7 | 0.01 |
|  | *Ostrinia furnacalis* | 0.3 (2) | 0.9 (26) | 1 | 1 | 11031 | 0.8 | 0.01 |
|  | *Palpita vitrealis* | N/A (1) | 1.4 (3) | 1 | 1 | 9598 | 0.6 | 1 |
|  | *Pygospila tyres* | 0.2 (6) | 0.2 (13) | 1 | 1 | 11098 | 0.0 | 0.94 |
|  | *Pyrausta sanguinalis* | 1.7 (5) | 1.7 (8) | 1 | 1 | 4623 | 0.0 | 0.14 |
|  | *Scirpophaga excerptalis* | 3.3 (16) | 3.2 (16) | 1 | 1 | 745 | 0.0 | 0.09 |
|  | *Scirpophaga incertulas* | 0 (3) | 2.7 (6) | 1 | 1 | 1863 | 0.4 | 0.08 |
|  | *Sitochroa palealis* | 0 (3) | 0.5 (18) | 1 | 1 | 11028 | 0.9 | 0.01 |
|  | *Sitochroa verticalis* | N/A (1) | 1.4 (5) | 1 | 1 | 5259 | 0.7 | 0.14 |
|  | *Spoladea recurvalis* | 0.8 (11) | 1.8 (124) | 1 | 1 | 18947 | 0.6 | 0.01 |
|  | *Synclera nr. traducalis* | N/A (1) | N/A | 1 | N/A | N/A | N/A | N/A |
|  | *Syngamia falsidicalis* | 0 (3) | 0.2 (3) | 1 | 1 | 4817 | 1.0 | 0.39 |
|  | *Udea exigualis* | N/A (1) | N/A | 1 | N/A | N/A | N/A | N/A |
|  | *Udea ferrugalis* | N/A (1) | 1.5 (7) | 1 | 1 | 10579 | 0.4 | 0.04 |
| Depressariidae | *Ethmia circumdatella* | 0 (2) | 9.6 (5) | 1 | 2 | 8784 | 0.0 | 0.39 |
|  | *Ethmia dodecea* | N/A (1) | 1.8 (4) | 1 | 1 | 5675 | 1.0 | 0.29 |
| Erebidae | *Acantholipes trimeni* | N/A (1) | 0.6 (8) | 1 | 1 | 7232 | 0.0 | 0.25 |
|  | *Achaea janata* | 0.2 (6) | 0.6 (44) | 1 | 1 | 15365 | 0.0 | 0.6 |
|  | *Aganais ficus* | 1.4 (9) | 1.4 (10) | 1 | 1 | 3031 | 0.0 | 0.88 |
|  | *Aloa lactinea* | 0 (11) | N/A | 1 | N/A | N/A | N/A | N/A |
|  | *Alphaea imbuta* | N/A (1) | N/A | 1 | N/A | N/A | N/A | N/A |
|  | *Amata cysseus* | N/A (1) | N/A | 1 | N/A | N/A | N/A | N/A |
|  | *Amerila astreus* | 0.6 (3) | 5.5 (6) | 1 | 2 | 4255 | 1.0 | 0.02 |
|  | *Andala unifascia* | 0.5 (11) | 0.5 (11) | 1 | 1 | 172 | 0.0 | 0.6 |
|  | *Anomis flava* | N/A (1) | 1.4 (32) | 1 | 1 | 18232 | 0.2 | 0.01 |
|  | *Anomis lyona* | 0 (2) | N/A | 1 | N/A | N/A | N/A | N/A |
|  | *Anumeta harteri* | 0.6 (5) | 0.6 (5) | 1 | 1 | 375 | 0.0 | 0.46 |
|  | *Arcte coerula* | N/A (1) | N/A | 1 | N/A | N/A | N/A | N/A |
|  | *Areas imperialis* | 0 (2) | N/A | 1 | N/A | N/A | N/A | N/A |
|  | *Asota caricae* | 0 (3) | 2.4 (64) | 1 | 1 | 10084 | 0.2 | 0.01 |
|  | *Calesia haemorrhoa* | N/A (1) | N/A | 1 | N/A | N/A | N/A | N/A |
|  | *Callindra principalis* | 0.5 (14) | 0.5 (14) | 1 | 1 | 189 | 0.0 | 0.09 |
|  | *Calliteara cerebosa* | 0.3 (8) | 0.3 (8) | 1 | 1 | 243 | 0.9 | 0.02 |
|  | *Calliteara grotei* | N/A (1) | N/A | 1 | N/A | N/A | N/A | N/A |
|  | *Carcinopyga lichenigera* | 0 (6) | N/A | 1 | N/A | N/A | N/A | N/A |
|  | *Casama vilis* | 0.2 (3) | N/A | 1 | N/A | N/A | N/A | N/A |
|  | *Catocala flavescens* | 0 (7) | N/A | 1 | N/A | N/A | N/A | N/A |
|  | *Catocala inconstans* | 0.2 (4) | 0.2 (4) | 1 | 1 | 55 | 0.0 | 1 |
|  | *Catocala pudica* | N/A (1) | N/A | 1 | N/A | N/A | N/A | N/A |
|  | *Chalciope mygdon* | N/A (1) | N/A | 1 | N/A | N/A | N/A | N/A |
|  | *Chrysopera combinans* | N/A (1) | 0.8 (5) | 1 | 1 | 9975 | 0.3 | 0.07 |
|  | *Chrysorabdia bivitta* | 0.2 (10) | 0.2 (10) | 1 | 1 | 172 | 0.0 | 0.64 |
|  | *Chrysorabdia viridana* | 0 (2) | N/A | 1 | N/A | N/A | N/A | N/A |
|  | *Cladarctia quadriramosa* | 0.6 (13) | 0.6 (13) | 1 | 1 | 172 | 0.0 | 0.52 |
|  | *Clytie devia* | 0.8 (4) | 0.8 (4) | 1 | 1 | 43 | 0.8 | 0.23 |
|  | *Clytie illunaris* | 0.3 (5) | 2.1 (8) | 1 | 2 | 5109 | 0.7 | 0.03 |
|  | *Creatonotos gangis* | 0.6 (16) | 1.8 (35) | 1 | 1 | 10868 | 0.9 | 0.01 |
|  | *Creatonotos transiens* | 0.5 (6) | 1.2 (9) | 1 | 1 | 4877 | 0.5 | 0.01 |
|  | *Cyana puella* | 0.5 (15) | 0.5 (15) | 1 | 1 | 438 | 0.0 | 0.8 |
|  | *Daona constellans* | 0.3 (2) | 2.1 (8) | 1 | 1 | 10292 | 0.4 | 0.07 |
|  | *Drasteria saisani* | 0 (2) | N/A | 1 | N/A | N/A | N/A | N/A |
|  | *Dysgonia algira* | 0.3 (3) | 3.7 (4) | 1 | 2 | 5315 | 1.0 | 0.14 |
|  | *Dysgonia rogenhoferi* | N/A (1) | N/A | 1 | N/A | N/A | N/A | N/A |
|  | *Dysgonia torrida* | N/A (1) | 1.6 (3) | 1 | 2 | 5439 | 1.0 | 0.37 |
|  | *Eilema palliatella* | 0.6 (3) | 0.6 (3) | 1 | 1 | 106 | 0.9 | 0.25 |
|  | *Episparis liturata* | N/A (1) | N/A | 1 | N/A | N/A | N/A | N/A |
|  | *Erebus albicinctus* | 0 (2) | N/A | 1 | N/A | N/A | N/A | N/A |
|  | *Erebus caprimulgus* | N/A (1) | 6.9 (5) | 1 | 3 | 4335 | 0.6 | 0.21 |
|  | *Ericeia subcinerea* | 0 (5) | N/A | 1 | N/A | N/A | N/A | N/A |
|  | *Eublemma nr. fastrei* | N/A (1) | N/A | 1 | N/A | N/A | N/A | N/A |
|  | *Eublemma ostrina* | 0.2 (2) | N/A | 1 | N/A | N/A | N/A | N/A |
|  | *Eublemma parva* | 0.5 (9) | 6.3 (14) | 1 | 2 | 5272 | 0.1 | 0.18 |
|  | *Eudocima materna* | N/A (1) | 0.2 (9) | 1 | 1 | 9771 | 0.0 | 0.33 |
|  | *Euproctis cervina* | 1.5 (23) | 1.5 (23) | 1 | 1 | 1061 | 0.3 | 0.01 |
|  | *Euproctis trispila* | N/A (1) | 1.1 (18) | 1 | 1 | 9672 | 0.9 | 0.05 |
|  | *Fodina stola* | 0.7 (10) | N/A | 1 | N/A | N/A | N/A | N/A |
|  | *Gesonia obeditalis* | N/A (1) | 2.1 (16) | 1 | 2 | 10089 | 0.0 | 0.08 |
|  | *Gnamptonyx innexa* | 0.5 (9) | 0.5 (18) | 1 | 1 | 5230 | 0.0 | 0.1 |
|  | *Gonitis sabulifera* | 0.5 (5) | 0.5 (5) | 1 | 1 | 225 | 0.1 | 0.86 |
|  | *Grammodes geometrica* | N/A (1) | N/A | 1 | N/A | N/A | N/A | N/A |
|  | *Grammodes stolida* | 0.8 (4) | 8.1 (5) | 1 | 2 | 1274 | 0.0 | 0.34 |
|  | *Heteropalpia robusta* | 0.2 (2) | N/A | 1 | N/A | N/A | N/A | N/A |
|  | *Hydrillodes metisalis* | 0.9 (15) | 5.8 (22) | 1 | 2 | 11027 | 0.2 | 0.01 |
|  | *Hypena laceratalis* | 0.2 (12) | 8.8 (24) | 1 | 4 | 10605 | 0.2 | 0.01 |
|  | *Hypena mandatalis* | 0.2 (3) | 9.8 (5) | 1 | 2 | 9512 | 1.0 | 0.07 |
|  | *Hypocala deflorata* | N/A (1) | 3.2 (26) | 1 | 2 | 11152 | 0.9 | 0.01 |
|  | *Hypocala nr. rostrata* | 0.5 (8) | 0.5 (8) | 1 | 1 | 669 | 0.5 | 0.07 |
|  | *Hypocala subsatura* | 0.9 (3) | 0.9 (5) | 1 | 1 | 5900 | 0.1 | 0.57 |
|  | *Hypotacha indecisa* | 1.1 (58) | 1.1 (58) | 1 | 1 | 325 | 0.0 | 0.54 |
|  | *Juxtarctia quadriguttata* | N/A (1) | N/A | 1 | N/A | N/A | N/A | N/A |
|  | *Lemyra stigmata* | 0.3 (3) | 0.3 (3) | 1 | 1 | 46 | 1.0 | 0.3 |
|  | *Lygephila craccae1 complex* | N/A (1) | N/A | 1 | N/A | N/A | N/A | N/A |
|  | *Lygephila craccae2 complex* | N/A (2) | N/A | 1 | N/A | N/A | N/A | N/A |
|  | *Lygephila dorsigera group* | 0 (4) | N/A | 1 | N/A | N/A | N/A | N/A |
|  | *Mocis proverai* | 0.3 (5) | 0.3 (5) | 1 | 1 | 96 | 0.0 | 0.39 |
|  | *Nebrarctia transversa* | 2.2 (22) | 2.1 (22) | 1 | 1 | 266 | 0.0 | 0.05 |
|  | *Ophiusa tirhaca* | 0.2 (2) | 2.7 (9) | 1 | 3 | 11525 | 0.3 | 0.02 |
|  | *Ophiusa triphaenoides* | N/A (1) | N/A | 1 | N/A | N/A | N/A | N/A |
|  | *Ophiusa umbrilinea* | N/A (1) | N/A | N/A | N/A | N/A | N/A | N/A |
|  | *Pandesma robusta* | 0.6 (32) | 0.6 (42) | 1 | 1 | 8339 | 0.0 | 0.12 |
|  | *Pericyma mendax* | N/A (1) | N/A | 1 | N/A | N/A | N/A | N/A |
|  | *Plecoptera reflexa* | 0.3 (21) | 0.3 (21) | 1 | 1 | 485 | 0.0 | 0.27 |
|  | *Polydesma boarmoides* | N/A (1) | N/A | 1 | N/A | N/A | N/A | N/A |
|  | *Rema tetraspila* | 0.2 (3) | 0.2 (3) | 1 | 1 | 160 | 1.0 | 0.37 |
|  | *Saroba ceylonica* | 0.2 (8) | 0.2 (8) | 1 | 1 | 99 | 0.0 | 0.89 |
|  | *Sidyma apicalis* | 0 (2) | N/A | 1 | N/A | N/A | N/A | N/A |
|  | *Sommeria hearseyana* | 0.6 (26) | 0.6 (26) | 1 | 1 | 433 | 0.0 | 0.34 |
|  | *Spilarctia casigneta group* | 0.5 (3) | 0.5 (3) | 1 | 1 | 23 | 1.0 | 0.29 |
|  | *Spilarctia casigneta2 group* | 0.5 (7) | N/A | 1 | N/A | 40 | 0.004 | 0.48 |
|  | *Spilarctia casigneta3 group* | 0.2 (2) | N/A | 1 | N/A | N/A | N/A | N/A |
|  | *Spilarctia leopardina* | 1.1 (15) | 1.1 (15) | 1 | 1 | 243 | 0.2 | 0.08 |
|  | *Spilosoma erythrozona* | 0.8 (17) | 0.8 (17) | 1 | 1 | 180 | 0.1 | 0.02 |
|  | *Spilosoma obliqua* | 0.0 (2) | 0.6 (3) | 1 | 1 | 508 | 1.0 | 0.3 |
|  | *Spilosoma xanthogaster* | 0.5 (5) | N/A | 1 | N/A | 57 | 0.05 | 0.33 |
|  | *Spirama helicina* | N/A (1) | N/A | 1 | N/A | N/A | N/A | N/A |
|  | *Tatargina pannosa* | 0.6 (12) | 0.6 (12) | 1 | 1 | 237 | 0.2 | 0.07 |
|  | *Trigonodes hyppasia* | 0 (3) | 10 (40) | 1 | 2 | 10959 | 0.0 | 0.07 |
|  | *Utetheisa lotrix* | N/A (1) | 1 (38) | 1 | 1 | 11053 | 0.4 | 0.03 |
|  | *Utetheisa nr. lotrix* | 1 (5) | 1 (5) | 1 | 1 | 39 | 0.0 | 0.62 |
|  | *Utetheisa pulchella* | 0.5 (8) | 0.6 (18) | 1 | 1 | 7992 | 0.0 | 0.38 |
|  | *Zethes insularis* | N/A (1) | N/A | 1 | N/A | N/A | N/A | N/A |
| Eupterotidae | *Eupterote undata* | N/A (1) | N/A | 1 | N/A | N/A | N/A | N/A |
|  | *Ganisa plana* | 0.5 (5) | 7.6 (7) | 1 | 2 | 3639 | 1.0 | 0.03 |
| Euteliidae | *Eutelia adulatricoides* | 0.2 (2) | N/A | 1 | N/A | N/A | N/A | N/A |
|  | *Lophoptera lineigera* | 0.3 (8) | 6.8 (12) | 1 | 2 | 4619 | 0.2 | 0.02 |
|  | *Odontodes aleuca* | 0.3 (20) | 0.3 (21) | 1 | 1 | 3070 | 0.0 | 0.49 |
| Gelechiidae | *Ephysteris promptella* | N/A (1) | 5.5 (35) | 1 | 3 | 11712 | 0.0 | 0.18 |
|  | *Pectinophora gossypiella* | 0 (5) | 0.2 (20) | 1 | 1 | N/A | N/A | N/A |
|  | *Sitotroga cerealella* | N/A (1) | 8.1 (3) | 1 | 2 | 7551 | 0.8 | 0.35 |
| Geometridae | *Abraxas leopardina* | N/A (1) | N/A | 1 | N/A | N/A | N/A | N/A |
|  | *Agathia lycaenaria* | 0 (2) | 2.6 (3) | 1 | 1 | 9732 | 1.0 | 0.34 |
|  | *Alcis depravata* | N/A (1) | N/A | 1 | N/A | N/A | N/A | N/A |
|  | *Alcis trikotaria* | 1.1 (11) | 1.1 (11) | 1 | 1 | 264 | 0.1 | 0.03 |
|  | *Amraica recursaria* | 0.5 (3) | N/A | 1 | N/A | N/A | N/A | N/A |
|  | *Apoheterolocha quadaria* | N/A (1) | N/A | 1 | N/A | N/A | N/A | N/A |
|  | *Archaeobalbis ochreipicta* | N/A (1) | N/A | 1 | N/A | N/A | N/A | N/A |
|  | *Ascotis selenaria* | 0.5 (2) | N/A | 1 | N/A | N/A | N/A | N/A |
|  | *Asthena albosignata* | N/A (1) | N/A | 1 | N/A | N/A | N/A | N/A |
|  | *Biston betularia* | 0.2 (8) | 4.4 (219) | 1 | 3 | 12278 | 0.2 | 0.01 |
|  | *Biston regalis* | 0 (10) | 6 (11) | 1 | 2 | 4661 | 1.0 | 0.1 |
|  | *Biston suppressaria* | 1.8 (8) | 1.8 (8) | 2 | 1 | 121 | 0.0 | 0.5 |
|  | *Chiasmia aestimaria* | 0.2 (3) | 2.6 (5) | 1 | 2 | 5616 | 0.8 | 0.08 |
|  | *Chiasmia hebesata* | 3.8 (2) | 7.2 (3) | 2 | 3 | N/A | N/A | N/A |
|  | *Chiasmia nora* | N/A (1) | N/A | 1 | N/A | N/A | N/A | N/A |
|  | *Chloroclystis ablechra* | N/A (1) | N/A | 1 | N/A | N/A | N/A | N/A |
|  | *Chlororithra fea* | N/A (1) | N/A | 1 | N/A | N/A | N/A | N/A |
|  | *Cleora fraterna* | N/A (1) | N/A | 1 | N/A | N/A | N/A | N/A |
|  | *Comibaena pictipennis* | N/A (1) | N/A | 1 | N/A | N/A | N/A | N/A |
|  | *Ctenognophos altissimus* | N/A (1) | N/A | 1 | N/A | N/A | N/A | N/A |
|  | *Cyclophora puppillaria* | N/A (1) | 0.6 (23) | 1 | 1 | 6717 | 0.7 | 0.03 |
|  | *Ectropis dentilineata* | N/A (1) | N/A | 1 | N/A | N/A | N/A | N/A |
|  | *Euphyia subangulata* | 0.3 (3) | 0.3 (3) | 1 | 1 | 58 | 1.0 | 0.3 |
|  | *Eupithecia interrubrescens* | N/A (1) | N/A | 1 | N/A | N/A | N/A | N/A |
|  | *Gnopharmia colchidaria* | N/A (1) | 0.6 (19) | 1 | 1 | 2308 | 0.0 | 0.2 |
|  | *Gnophos bidentatus* | N/A (1) | N/A | 1 | N/A | N/A | N/A | N/A |
|  | *Hemidromodes sabulifera hessa* | N/A (1) | 2.8 (7) | 1 | 1 | 3456 | 0.4 | 0.03 |
|  | *Hemistola chrysoprasaria lissas* | 0.2 (5) | 0.2 (5) | 1 | 1 | 88 | 0.0 | 0.44 |
|  | *Hemithea punctifimbria* | 0.8 (6) | 0.8 (6) | 1 | 1 | 716 | 0.0 | 0.53 |
|  | *Heterolocha phoenicotaeniata* | N/A (1) | N/A | 1 | N/A | N/A | N/A | N/A |
|  | *Hydatocapnia gemina* | N/A (1) | N/A | 1 | N/A | N/A | N/A | N/A |
|  | *Idaea eremica* | 0.2 (4) | 2.4 (7) | 1 | 2 | 1993 | 1.0 | 0.01 |
|  | *Isturgia disputaria* | 1.1 (17) | 1.1 (24) | 1 | 1 | 5241 | 0.2 | 0.01 |
|  | *Lassaba parvalbidaria* | N/A (1) | N/A | 1 | N/A | N/A | N/A | N/A |
|  | *Microloxia herbaria* | N/A (1) | 2 (12) | 1 | 2 | 5439 | 0.1 | 0.11 |
|  | *Microloxia indecretata* | N/A (1) | 0.3 (3) | 1 | 1 | 1490 | 0.2 | 0.68 |
|  | *Odontopera kametaria* | 0.6 (10) | 0.6 (10) | 1 | 1 | 97 | 0.1 | 0.23 |
|  | *Odontopera muscularia* | 2.3 (9) | 2.3 (9) | 1 | 1 | 175 | 0.0 | 0.84 |
|  | *Opisthograptis luteolata* | 0.5 (4) | 8 (31) | 1 | 2 | 5721 | 0.9 | 0.01 |
|  | *Ourapteryx nepalensis* | N/A (1) | N/A | 1 | N/A | N/A | N/A | N/A |
|  | *Oxymacaria temeraria* | N/A (1) | N/A | 1 | N/A | N/A | N/A | N/A |
|  | *Pelagodes aucta* | N/A (1) | N/A | 1 | N/A | N/A | N/A | N/A |
|  | *Phaiogramma discessa* | 0.8 (2) | 0.9 (5) | 1 | 1 | 1775 | 0.0 | 0.48 |
|  | *Photoscotosia miniosata* | 0 (2) | N/A | 1 | N/A | N/A | N/A | N/A |
|  | *Plagodis reticulata* | N/A (1) | N/A | 1 | N/A | N/A | N/A | N/A |
|  | *Problepsis ocellata* | 0.5 (4) | 0.5 (4) | 1 | 1 | 129 | 0.3 | 0.25 |
|  | *Rhodometra sacraria* | 1.4 (6) | 2.4 (34) | 1 | 1 | 8305 | 0.2 | 0.01 |
|  | *Scopula emissaria* | N/A (1) | 1.7 (5) | 1 | 3 | 9752 | 0.9 | 0.03 |
|  | *Scopula minorata* | 0.3 (6) | 4.4 (24) | 1 | 2 | 8241 | 0.3 | 0.01 |
|  | *Scopula subpunctaria* | 1.4 (5) | 7.3 (13) | 1 | 2 | 8222 | 0.4 | 0.03 |
|  | *Scopula terminata machadoi* | N/A (1) | N/A | 1 | N/A | N/A | N/A | N/A |
|  | *Somatina anthophilata* | 0.3 (2) | N/A | 1 | N/A | N/A | N/A | N/A |
|  | *Thinopteryx crocoptera* | N/A (1) | N/A | 1 | N/A | N/A | N/A | N/A |
|  | *Traminda mundissima* | 0.6 (7) | 10.2 (45) | 1 | 2 | 11989 | 0.9 | 0.01 |
|  | *Xanthorhoe saturata* | 0.5 (3) | 0.5 (3) | 1 | 1 | 57 | 1.0 | 0.31 |
| Gracillariidae | *Stomphastis dodonaea* | N/A (1) | N/A | 1 | N/A | N/A | N/A | N/A |
| Hyblaeidae | *Hyblaea puera* | 0 (2) | 1.5 (5) | 1 | 3 | 16074 | 0.7 | 0.11 |
| Lasiocampidae | *Chilena sordida* | 0.8 (3) | 0.8 (3) | 1 | 1 | 1123 | 1.0 | 0.3 |
|  | *Estigena encausta* | 0.2 (15) | 0.2 (15) | 1 | 1 | 482 | 0.0 | 0.84 |
|  | *Similodora fia* | 1.2 (3) | 1.2 (3) | 1 | 1 | 149 | 0.9 | 0.3 |
|  | *Streblote alpherakyi* | 0 (2) | N/A | 1 | N/A | N/A | N/A | N/A |
|  | *Streblote helpsi* | 0.9 (2) | 0.9 (3) | 1 | 1 | 4487 | 0.8 | 1 |
|  | *Trabala vishnou* | 0.3 (8) | N/A | 1 | N/A | N/A | N/A | N/A |
| Limacodidae | *Aphendala flavina* | 0.2 (2) | N/A | 1 | N/A | N/A | N/A | N/A |
|  | *Altha nivea* | 0.0 (5) | 10.8 (7) | 1 | 2 | 5455 | 1.0 | 0.04 |
| Noctuidae | *Acontia lucida* | 0.3 (2) | 0.3 (3) | 1 | 1 | 3579 | 0.3 | 1 |
|  | *Acontia opalinoides* | N/A (1) | N/A | 1 | N/A | N/A | N/A | N/A |
|  | *Acronicta rubiginosa* | N/A (1) | N/A | 1 | N/A | N/A | N/A | N/A |
|  | *Actinotia intermediata* | N/A (1) | 0.6 (3) | 1 | 1 | 5833 | 1.0 | 0.37 |
|  | *Aedia leucomelas* | 1.1 (3) | 10.5 (23) | 1 | 3 | 16281 | 0.9 | 0.01 |
|  | *Agrotis ipsilon* | 0.5 (33) | 1.4 (175) | 1 | 1 | 18894 | 0.4 | 0.01 |
|  | *Agrotis munda* | 0.8 (17) | 2.8 (210) | 1 | 2 | 14167 | 0.8 | 0.01 |
|  | *Agrotis segetum* | 0.3 (15) | 1.3 (45) | 1 | 1 | 9615 | 0.0 | 0.15 |
|  | *Amyna axis* | 2.3 (11) | 5 (39) | 2 | 3 | 17496 | 0.4 | 0.01 |
|  | *Amyna punctum* | 0.3 (14) | 4.6 (21) | 1 | 3 | 11809 | 0.9 | 0.01 |
|  | *Anarta trifolii* | 0.2 (2) | 1.6 (26) | 1 | 1 | 12950 | 0.1 | 0.03 |
|  | *Antoculeora ornatissima* | N/A (1) | 3.8 (3) | 1 | 2 | 5767 | 1.0 | 0.36 |
|  | *Apamea lateritia* | 0.2 (2) | 1.7 (7) | 1 | 2 | 5272 | 0.8 | 0.04 |
|  | *Athetis lepigone* | 0.2 (5) | 5 (6) | 1 | 2 | 4957 | 1.0 | 0.17 |
|  | *Autographa nigrisigna* | 0 (10) | N/A | 1 | N/A | N/A | N/A | N/A |
|  | *Axylia putris* | 0.5 (8) | 5.8 (29) | 1 | 2 | 8781 | 0.5 | 0.01 |
|  | *Callopistria repleta* | N/A (1) | N/A (2) | 1 | 1 | N/A | N/A | N/A |
|  | *Chrysodeixis acuta* | 0 (7) | 0.8 (25) | 1 | 1 | 14845 | 0.4 | 0.01 |
|  | *Chrysodeixis eriosoma* | 0.3 (14) | 0.4 (38) | 1 | 1 | 11500 | 0.6 | 0.01 |
|  | *Condica capensis* | N/A (1) | 1.1 (14) | 1 | 1 | 11738 | 0.8 | 0.01 |
|  | *Condica illecta* | 0.5 (24) | 1 (58) | 1 | 1 | 16454 | 0.3 | 0.01 |
|  | *Cryphia raptricula* | N/A (1) | N/A | 1 | N/A | N/A | N/A | N/A |
|  | *Ctenoplusia albostriata* | 0.2 (4) | 0.6 (29) | 1 | 1 | 10984 | 0.0 | 0.46 |
|  | *Ctenoplusia furcifera ogovana* | 0 (3) | N/A | 1 | N/A | N/A | N/A | N/A |
|  | *Cucullia nigrifascia* | N/A (1) | N/A | 1 | N/A | N/A | N/A | N/A |
|  | *Diarsia hoenei* | 0 (4) | N/A | 1 | N/A | N/A | N/A | N/A |
|  | *Diarsia nigrosigna* | 0 (2) | N/A | 1 | N/A | N/A | N/A | N/A |
|  | *Dichagyris celsicola* | 0 (2) | N/A | 1 | N/A | N/A | N/A | N/A |
|  | *Euxoa agricola* | N/A (1) | N/A | 1 | N/A | N/A | N/A | N/A |
|  | *Hadena compta* | N/A (1) | 1.4 (9) | 1 | 1 | 5100 | 0.7 | 0.02 |
|  | *Hecatera dysodea* | N/A (1) | 1.8 (12) | 1 | 2 | 5570 | 1.0 | 0.04 |
|  | *Helicoverpa armigera* | 1.1 (64) | 2 (337) | 1 | 1 | 19284 | 0.0 | 0.01 |
|  | *Heliocheilus confertissima* | 0.5 (6) | 0.5 (6) | 1 | 1 | 168 | 0.1 | 1 |
|  | *Heliothis peltigera* | 0.2 (4) | 0.3 (10) | 1 | 1 | 5689 | 0.0 | 0.24 |
|  | *Leucania loreyi* | 0.6 (12) | 0.9 (24) | 1 | 1 | 19665 | 0.1 | 0.03 |
|  | *Leucania venalba* | 0.3 (3) | 5.7 (5) | 1 | 2 | 8684 | 1.0 | 0.08 |
|  | *Maliattha signifera* | 0.2 (4) | 6.5 (18) | 1 | 2 | 10967 | 0.0 | 0.25 |
|  | *Mamestra brassicae* | 0 (2) | 2.6 (51) | 1 | 2 | 8051 | 0.9 | 0.01 |
|  | *Mythimna ferrago* | N/A (1) | 4.3 (28) | 1 | 2 | 5713 | 0.1 | 0.06 |
|  | *Mythimna l-album* | N/A (1) | 0.2 (17) | 1 | 1 | 5544 | 0.0 | 0.62 |
|  | *Mythimna languida* | 0.2 (2) | 2.8 (4) | 1 | 2 | 5564 | 0.9 | 0.37 |
|  | *Mythimna separata* | 0.4 (9) | 0.6 (24) | 1 | 1 | 10705 | 0.0 | 0.05 |
|  | *Peridroma saucia* | 0 (14) | 0.4 (78) | 1 | 1 | 13053 | 0.0 | 0.74 |
|  | *Pseudozarba bipartita* | N/A (1) | N/A | 1 | N/A | N/A | N/A | N/A |
|  | *Pyrrhia nr. umbra* | N/A (1) | N/A | 1 | N/A | N/A | N/A | N/A |
|  | *Rusicada combinans* | N/A (1) | 6.7 (3) | 1 | 2 | 4245 | 1.0 | 0.49 |
|  | *Sesamia inferens* | 1.2 (4) | 5.2 (6) | 1 | 3 | 1772 | 0.5 | 0.08 |
|  | *Spodoptera cilium* | 0.2 (14) | 0.3 (19) | 1 | 1 | 8188 | 0.6 | 0.01 |
|  | *Spodoptera exigua* | 0.6 (21) | 5.4 (266) | 1 | 2 | 18215 | 0.4 | 0.01 |
|  | *Spodoptera littoralis* | 0.2 (2) | N/A | 1 | N/A | N/A | N/A | N/A |
|  | *Spodoptera litura* | 0.7 (25) | 0.8 (89) | 1 | 1 | 11168 | 0.0 | 0.04 |
|  | *Spodoptera pecten* | N/A (1) | N/A | 1 | N/A | N/A | N/A | N/A |
|  | *Thysanoplusia intermixta* | 0 (2) | N/A | 1 | N/A | N/A | N/A | N/A |
|  | *Thysanoplusia orichalcea* | 0.3 (20) | 0.6 (41) | 1 | 1 | 12557 | 0.0 | 0.02 |
|  | *Trichoplusia daubei* | N/A (1) | N/A | 1 | N/A | N/A | N/A | N/A |
|  | *Trichoplusia ni* | 0 (7) | 4.8 (37) | 1 | 2 | 13827 | 0.9 | 0.01 |
|  | *Xanthodes brunnescens* | 1.4 (4) | N/A | 1 | N/A | N/A | N/A | N/A |
|  | *Xestia c-nigrum* | 0.3 (23) | 1.4 (201) | 1 | 1 | 12548 | 0.1 | 0.01 |
| Nolidae | *Aquis orbicularis* | 0.3 (4) | 0.3 (4) | 1 | 1 | 134 | 0.3 | 0.85 |
|  | *Earias insulana* | 0.3 (7) | 0.3 (12) | 1 | 1 | 7993 | 0.0 | 0.81 |
|  | *Earias luteolaria* | N/A (1) | 1.2 (11) | 1 | 1 | 10969 | 0.9 | 0.09 |
|  | *Earias vittella* | 0.2 (4) | 2 (10) | 1 | 1 | 9985 | 0.0 | 0.39 |
|  | *Garella ruficirra* | N/A (1) | N/A (2) | 1 | 1 | N/A | N/A | N/A |
|  | *Nola analis* | N/A (1) | N/A (2) | 1 | 1 | N/A | N/A | N/A |
|  | *Nola mesoscia* | 0.3 (2) | N/A | 1 | N/A | N/A | N/A | N/A |
|  | *Risoba prominens* | N/A (1) | N/A (2) | 1 | 1 | N/A | N/A | N/A |
| Notodontidae | *Acmeshachia gigantea* | 0 (2) | N/A | 1 | N/A | N/A | N/A | N/A |
|  | *Antheua servula* | 1.9 (2) | N/A | 1 | N/A | N/A | N/A | N/A |
|  | *Gazalina apsara* | 0 (10) | N/A | 1 | N/A | N/A | N/A | N/A |
|  | *Gazalina chrysolopha* | 0.6 (16) | N/A | 1 | N/A | N/A | N/A | N/A |
|  | *Harpyia longipennis* | N/A (1) | N/A | 1 | N/A | N/A | N/A | N/A |
|  | *Kamalia kandyia ronii* | 0.2 (2) | N/A | 1 | N/A | N/A | N/A | N/A |
|  | *Neopheosia fasciata* | 0 (2) | 3.4 (3) | 1 | 2 | 5750 | 1.0 | 0.28 |
|  | *Phalera combusta* | 0 (2) | N/A | 1 | N/A | N/A | N/A | N/A |
|  | *Phalera parivala* | N/A (1) | N/A | 1 | N/A | N/A | N/A | N/A |
|  | *Rachia striata* | N/A (1) | N/A | 1 | N/A | N/A | N/A | N/A |
|  | *Semidonta nigribasis* | 0.9 (4) | 0.9 (4) | 1 | 1 | 161 | 1.0 | 0.21 |
|  | *Syntypistis umbrosa stauropoides* | 0 (2) | 0.3 (3) | 1 | 1 | 4538 | 1.0 | 0.33 |
| Plutellidae | *Leuroperna sera* | N/A (1) | 1.6 (20) | 1 | 1 | 11048 | 0.9 | 0.06 |
|  | *Plutella xylostella* | 2 (5) | 3 (895) | 1 | 1 | N/A | N/A | N/A |
| Psychidae | *Eumeta crameri* | N/A (1) | N/A | 1 | N/A | N/A | N/A | N/A |
| Pterophoridae | *Emmelina monodactyla* | N/A (1) | 16.3 (79) | 1 | 4 | 12100 | 0.2 | 0.01 |
| Pyralidae | *Aglossa aglossalis* | 0 (2) | 2.1 (6) | 1 | 1 | 3325 | 1.0 | 0.07 |
|  | *Ancylodes pallens* | N/A (1) | N/A | 1 | N/A | N/A | N/A | N/A |
|  | *Cadra figulilella* | N/A (1) | 1.1 (4) | 1 | 1 | 12991 | 0.1 | 0.5 |
|  | *Calguia defiguralis* | N/A (1) | 10.2 (14) | 1 | 2 | 10969 | 0.8 | 0.01 |
|  | *Ceutholopha isidis* | 0.5 (7) | 0.5 (7) | 1 | 1 | 156 | 0.1 | 0.99 |
|  | *Endotricha olivacealis* | 1.1 (24) | 1.1 (24) | 1 | 1 | 207 | 0.4 | 0.02 |
|  | *Etiella zinckenella* | 1.7 (2) | 5.2 (19) | 1 | 3 | 15970 | 0.8 | 0.01 |
|  | *Hypsopygia mauritialis* | 0 (4) | 2.9 (10) | 1 | 2 | 10403 | 0.5 | 0.01 |
|  | *Lamoria anella* | 0.6 (2) | N/A | 1 | N/A | N/A | N/A | N/A |
|  | *Phycita arabica* | N/A (1) | N/A (2) | 1 | 1 | N/A | N/A | N/A |
|  | *Plodia interpunctella* | 0 (4) | 1.9 (29) | 1 | 1 | 16767 | 0.0 | 0.48 |
|  | *Sciota insignella* | 0.5 (13) | 0.5 (13) | 1 | 1 | 477 | 0.0 | 0.67 |
| Saturniidae | *Actias selene* | 0.5 (7) | 7.1 (13) | 1 | 2 | 4749 | 0.9 | 0.01 |
|  | *Rinaca lindia* | 1.1 (10) | 1.1 (10) | 1 | 1 | 22 | 0.0 | 0.46 |
|  | *Samia canningi* | 0 (3) | N/A | 1 | N/A | N/A | N/A | N/A |
| Scythrididae | *Eretmocera impactella* | N/A (1) | N/A | 1 | N/A | N/A | N/A | N/A |
| Sesiidae | *Sesia ommatiaeformis* | N/A (1) | N/A | 1 | N/A | N/A | N/A | N/A |
| Sphingidae | *Acherontia lachesis* | 0.0 (2) | 0.6 (3) | 1 | 1 | 8683 | 0.9 | 1 |
|  | *Acherontia styx* | 0.3 (7) | 0.8 (8) | 1 | 1 | 605 | 0.3 | 0.14 |
|  | *Acosmeryx naga hissarica* | 1.2 (14) | 1.2 (14) | 1 | 1 | 178 | 0.0 | 0.39 |
|  | *Agnosia orneus* | 0 (7) | N/A | 1 | N/A | N/A | N/A | N/A |
|  | *Agrius convolvuli* | 0.4 (15) | 5.1 (218) | 1 | 2 | 18013 | 0.7 | 0.01 |
|  | *Ambulyx schauffelbergeri* | N/A (1) | 1.8 (3) | 1 | 2 | 5809 | 0.0 | 0.64 |
|  | *Ampelophaga khasiana* | 0.3 (7) | 1.1 (8) | 1 | 1 | 3349 | 0.9 | 0.1 |
|  | *Anambulyx elwesi* | 0.5 (10) | 1.7 (11) | 1 | 1 | 3379 | 0.8 | 0.08 |
|  | *Cechenena lineosa* | 0.2 (9) | 1.2 (10) | 1 | 1 | 4388 | 1.0 | 0.08 |
|  | *Cechenena mirabilis* | 0.3 (8) | 0.3 (8) | 1 | 1 | 134 | 0.1 | 0.2 |
|  | *Cephonodes hylas* | 0.5 (4) | 0.8 (9) | 1 | 1 | 5688 | 0.0 | 0.73 |
|  | *Clanidopsis exusta* | 0.2 (10) | 0.5 (11) | 1 | 1 | 1484 | 0.7 | 0.11 |
|  | *Clanis deucalion* | 0.3 (2) | 0.7 (3) | 1 | 1 | 1151 | 0.3 | 0.24 |
|  | *Clanis phalaris* | N/A (1) | N/A | 1 | N/A | N/A | N/A | N/A |
|  | *Daphnis nerii* | 0 (4) | 0.3 (12) | 1 | 1 | 7332 | 0.1 | 0.87 |
|  | *Deilephila rivularis* | 0.0 (19) | 0.0 (19) | 1 | 1 | N/A | N/A | N/A |
|  | *Dolbina grisea* | 0.5 (12) | 0.6 (13) | 1 | 1 | 458 | 0.4 | 0.01 |
|  | *Dolbina inexacta* | N/A (1) | N/A | 1 | N/A | N/A | N/A | N/A |
|  | *Hippotion boerhaviae* | N/A (1) | 0.8 (17) | 1 | 1 | 11393 | 0.1 | 0.01 |
|  | *Hippotion celerio* | N/A (1) | 1.6 (96) | 1 | 1 | 17517 | 0.6 | 0.01 |
|  | *Hippotion rosetta* | N/A (1) | 1.0 (33) | 1 | 1 | 10903 | 0.0 | 0.51 |
|  | *Hyles chuvilini* | N/A (1) | N/A (4) | ACE9998 | 1 | N/A | N/A | N/A |
|  | *Hyles gallii* | N/A (1) | 1.7 (23) | 1 | 1 | 10513 | 0.1 | 0.01 |
|  | *Hyles hippophaes* | 0.4 (2) | 2.4 (5) | 1 | 3 | 5642 | 0.2 | 0.03 |
|  | *Hyles livornica* | 0.9 (10) | 0.9 (15) | 1 | 1 | 8421 | 0.1 | 0.11 |
|  | *Hyles stroehlei* | 0.0 (2) | 0.3 (7) | ACE9998 | 1 | 277 | 0.0 | 0.43 |
|  | *Leucophlebia emittens* | 0.3 (10) | N/A | 1 | N/A | N/A | N/A | N/A |
|  | *Leucophlebia lineata* | N/A (1) | N/A | 1 | N/A | N/A | N/A | N/A |
|  | *Macroglossum belis* | N/A (1) | N/A | 1 | N/A | N/A | N/A | N/A |
|  | *Macroglossum nycteris* | 0 (14) | N/A | 1 | N/A | N/A | N/A | N/A |
|  | *Macroglossum stellatarum* | 0.6 (4) | 0.8 (20) | 1 | 1 | 6192 | 0.0 | 0.28 |
|  | *Marumba dyras* | N/A (1) | N/A (2) | 1 | 2 | N/A | N/A | N/A |
|  | *Nephele hespera* | 0.3 (18) | 2.3 (29) | 1 | 2 | 10882 | 0.8 | 0.03 |
|  | *Polyptychus dentatus* | 0 (3) | N/A | 1 | N/A | N/A | N/A | N/A |
|  | *Polyptychus trilineatus* | 0 (6) | N/A | 1 | N/A | N/A | N/A | N/A |
|  | *Psilogramma increta* | 0 (2) | 3.8 (3) | 1 | 2 | 4327 | 1.0 | 0.41 |
|  | *Psilogramma vates* | 0.6 (3) | 0.6 (3) | 1 | 1 | 124 | 0.3 | 0.36 |
|  | *Rhagastis olivacea* | 0 (2) | N/A | 1 | N/A | N/A | N/A | N/A |
|  | *Sataspes tagalica* | N/A (1) | N/A | 1 | N/A | N/A | N/A | N/A |
|  | *Smerinthus kindermannii* | 0 (2) | N/A (3) | 1 | 2 | N/A | N/A | N/A |
|  | *Theretra alecto* | 0 (5) | 2.2 (7) | 1 | 2 | 8755 | 0.4 | 0.16 |
|  | *Theretra oldenlandiae* | 0.9 (14) | 0.9 (20) | 1 | 1 | 4621 | 0.0 | 0.74 |
| Thyrididae | *Picrostomastis marginepunctalis* | N/A (1) | 1.7 (3) | 1 | 1 | 9450 | 1.0 | 0.26 |
| Tineidae | *Monopis monacha* | N/A (1) | 5.4 (3) | 1 | 2 | 9775 | 0.07 | 0.49 |
| Tortricidae | *Archips machlopis* | 0.8 (16) | 0.8 (16) | 1 | 1 | 112 | 0.0 | 0.7 |
|  | *Bactra blepharopis* | 0 (3) | 7.3 (10) | 1 | 2 | 10969 | 0.0 | 0.3 |
|  | *Bactra venosana* | 0.9 (18) | 7.4 (41) | 1 | 3 | 14171 | 0.8 | 0.01 |
|  | *Crocidosema plebejana* | N/A (1) | 6.9 (120) | 1 | 4 | 18445 | 0.9 | 0.01 |
|  | *Cydia nr. Choleropa* | 0 (2) | N/A | 1 | N/A | N/A | N/A | N/A |
|  | *Dudua siderea* | N/A (1) | N/A (2) | 1 | 1 | N/A | N/A | N/A |
|  | *Epiblema foenella* | 0 (2) | 2.2 (23) | 1 | 1 | 9523 | 0.4 | 0.01 |
|  | *Grapholita delineana* | 0.5 (10) | 0.5 (11) | 1 | 1 | 5899 | 0.1 | 0.28 |
|  | *Lumaria probolias* | 0.8 (21) | N/A | 1 | N/A | N/A | N/A | N/A |
|  | *Rhopobota naevana* | N/A (1) | 3.8 (456) | 1 | 1 | 11022 | 0.1 | 0.01 |
| Yponomeutidae | *Yponomeuta evonymella* | 0.2 (11) | 1.4 (19) | 1 | 1 | 5492 | 0.9 | 0.01 |
| Zygaenidae | *Epizygaenella caschmirensis* | N/A (1) | N/A | 1 | N/A | N/A | N/A | N/A |

Pak = records from Pakistan; Comb = combined records from Pakistan and other regions; Geo. dist. = geographical distance in kilometers; M-R^2^ = Mantel test (Mantel et al. 1967) R-square; M-P = Mantel test P-value; N/A = not available or not calculated due to insufficient data.
